# Beyond balance: The role of the Vestibular system in action recognition

**DOI:** 10.1016/j.heliyon.2024.e38019

**Published:** 2024-09-18

**Authors:** Roberto Gammeri, Maria-Chiara Villa, Tommaso Ciorli, Anna Berti, Raffaella Ricci

**Affiliations:** a SAN (Space, Attention and actioN) Lab, Department of Psychology, University of Turin, Via Verdi, 10, Torino, 10124, Italy; b SAMBA (SpAtial, Motor and Bodily Awareness) Research Group, Department of Psychology, University of Turin, Via Verdi 10, Torino, 10124, Italy; c BIP (BraIn Plasticity and Behaviour Changes) Research Group, Department of Psychology, University of Turin, Italy

**Keywords:** Galvanic Vestibular stimulation, Vestibular system, Action recognition, Motor familiarity, Visual sensitivity, Mirror processing

## Abstract

**Background:**

Action recognition is a fundamental aspect of human interaction. This process is mediated by the activation of shared sensorimotor representations during action execution and observation. Although complex movements involving balance or head and trunk rotations require vestibular signals for effective execution, their role in the recognition of others' actions is still unknown.

**Objective:**

To investigate the causal involvement of the vestibular system in the discrimination of actions performed by others and whether this is influenced by motor familiarity.

**Methods:**

In a single-blind design involving 25 healthy participants, Galvanic Vestibular Stimulation (GVS) was administered during an Action Discrimination Task (ADT), in which videos of actions categorized as vestibular/non-vestibular and familiar/unfamiliar were presented. Following each video, participants were required to identify the climax of the previously viewed action between two image options, using a two-alternative forced choice paradigm. The ADT was performed in active and sham GVS conditions, with left or right anodal montages. Response Times (RTs), Accuracy, and subjective motor familiarity were recorded for each action category.

**Results:**

In sham GVS condition, an overall familiarity effect was observed, where RTs for familiar actions were faster than RTs for unfamiliar ones, regardless of vestibular engagement (*p* < *.001;* η_p_^2^ = .80). Conversely, under active GVS, a selective interference of the identification of vestibular familiar actions was observed compared to sham. Specifically, GVS prolonged RTs for recognizing familiar vestibular actions (*p = .004,* d = .59) while concurrently enhancing visual sensitivity (*d’*) for the same actions (*p = .03,* r = .21).

**Conclusion:**

These findings demonstrate the contribution of the vestibular system to action recognition. GVS disrupted the sensorimotor representation of vestibular actions and led to increased reliance on an alternative processing system focused on visual analysis of limb positions. This dissociation provides valuable insights for future investigations into the complex relationship between vestibular signals and cognitive processes involved in action identification, essential for developing innovative GVS interventions, particularly for individuals with sensorimotor or vestibular disorders.

## Highlights

• GVS selectively slows down the recognition of vestibular actions.
• GVS enhances visual sensitivity for vestibular actions.
• GVS impact on recognition times is lower in people relying more on visual information.
• Disrupting sensorimotor representations increases the weight of visual features.
• The vestibular system contributes to mirror mechanisms underlying action recognition.

## 1 Introduction

Action understanding is a basic social skill and a fundamental aspect of human interaction and social cognition. It enables us to interpret, predict and respond appropriately to the movements of others [[1], [2], [3]]. The cognitive processes involved in action understanding include the identification of the type of action (based on the configuration of body parts), its goal, and the actor's underlying intention [4]. The Mirror Neuron System (MNS) is one of the neural mechanisms believed to contribute significantly to action understanding, particularly in relation to action identification. It involves neurons in frontoparietal regions that are activated both when individuals perform a specific action and when they observe the same action performed by others [5]. According to the mirror neuron theory [6,7], when we observe an action, we activate an internal motor representation as if we were performing it. Therefore, a common neural representation would be activated during action execution and perception [8,9]. In support of this theory, altered brain activity in the areas involved in the 10.13039/100018306MNS has been shown to impair individuals' ability to perceive and understand others' actions [10]. Moreover, the individual's motor familiarity with the observed action strongly influences mirror processes. The observation of actions characterized by a high degree of motor familiarity is associated with increased activation of MNS regions [11,12] and enhanced performance in tasks involving action recognition [[13], [14], [15], [16]]. Therefore, the motor representation of familiar actions holds fundamental importance in the perceptual processing of analogous actions executed by others (for a review, see Ref. [17]). Furthermore, complex movements, such as those necessitating balance control or extensive head and trunk rotations, involve the vestibular system to be satisfactorily produced. Therefore, another factor to be considered in the identification of others' actions is the potential involvement of vestibular information. For instance, it has been shown that vestibular signals encoding linear and angular accelerations [18] play a crucial role not only in ensuring balance and posture but also in the perception of self-motion (i.e., perception of rotation, linear motion, path, and direction [19,20]). Interestingly, the ability to detect self-motion and control balance is strongly influenced by the movements of others [21]. For example, observing the body of others rotating in the opposite direction to one's own body impairs self-motion perception [22]. Consistently, the cortical projections of the vestibular system [23] to the supramarginal gyrus (SMG), the superior temporal sulcus (STS), the inferior parietal lobe (IPL), and the inferior frontal gyrus (IFG), partially overlap with areas showing mirror-like activity [24,25]. Despite its recognized role in the perception of self-movements [19,26] the possible involvement of the vestibular system in the construction of internal sensorimotor representations underlying the identification of others' actions is still unknown.

In the present study, we asked whether the vestibular system contributes to the recognition of others’ actions by employing Galvanic Vestibular Stimulation (GVS) to transiently modulate the vestibular input in healthy individuals. GVS is a non-invasive neuromodulation technique whereby weak electrical currents are applied to the vestibular nerve to modulate the transmission to the brain of vestibular signals encoded by peripheral organs (i.e., otoliths and semicircular canals). We specifically investigated whether disruption of vestibular input by supra-threshold GVS might affect the recognition of actions whose correct execution strongly depends on the vestibular activation. For example, maintaining balance on one foot necessitates the integration of proprioceptive, motor and visual signals with those arising from both the otoliths and semicircular canals, which sense linear and angular accelerations of the head and trunk, and enable rapid adjustments to maintain stability. Conversely, moving an upper limb independently of head and body movements does not significantly engage the vestibular system except for the estimation of static orientation relative to gravity, and can therefore be performed primarily through proprioceptive and visual signals. Participants performed a novel action discrimination task, with actions involving varying degrees of vestibular activity, during active or sham GVS. If vestibular information contributes to action recognition, the discrimination of actions that strongly depend on the vestibular control should be impaired by GVS compared to actions that do not depend primarily on this system. On the other hand, if vestibular information is not necessary for action recognition, we should not observe any difference between vestibular and non-vestibular actions. Furthermore, since familiarity is a relevant factor for the identification of actions, we also analyze its putative effects and interaction with GVS on vestibular and non-vestibular actions. It has been argued that the motor familiarity effect could result from predictive simulations through internal models of the actions (i.e., neural systems that allow the simulation of motor outcomes and the prediction of sensory consequences of an action). According to this hypothesis, when we observe familiar actions performed by others, we use the predictive models that we usually rely on when performing the same actions, and this allows us to simulate that action more accurately through a matching mechanism that activates previously stored motor representations [12,27]. By contrast, the identification of unfamiliar actions relies on different mechanisms [28]. We thus expected to observe overall faster performance in discriminating familiar versus unfamiliar actions, confirming the hypothesis that motor familiarity improves action recognition. In addition, since the vestibular system contributes to the computation of internal models of actions requiring strong vestibular activity, we might expect stronger GVS effects on vestibular actions that are also familiar, as they are expected to trigger more mirror-like processing.

## 2 Methods

### 2.1 Participants

A preliminary power analysis performed with G∗Power version 3.1.9.7 [29] to ascertain the minimum sample size needed to test the study's hypothesis showed that a sample size of N = 30 was necessary to achieve 90 % statistical power for detecting a medium effect size (Cohen's f = .25) at a significance level of α = .05, using a repeated measure ANOVA. Thirty-five healthy voluntary participants were initially recruited for this study, following specific exclusion criteria (see Appendix A). Data on participants' educational levels, sports engagement, and training frequency were registered. Three participants were excluded from the study due to a vestibular threshold exceeding 2 mA [30]. A final sample of 32 healthy individuals participated in the study. Participants gave written informed consent to participate in the study, which was approved by the Ethical Committee of the University of Turin with the approval number 0212045.

### 2.2 Stimuli

Stimuli were specifically created for this study, inspired by the work of Calvo-Merino and colleagues [11]. They consisted of 3-s colorful videoclips (size: 1920 × 1080) presented at the center of a 24″ laptop screen (Asus VivoBook Pro N580V). Each video displayed an actor (either a man or a woman) performing different categories of actions in a full-body, third-person perspective view. Categories were specifically generated depending on vestibularity and familiarity differences. With *vestibularity* we refer to the degree of involvement of the vestibular system in executing a given action: actions involving balance or head and trunk rotations entail a substantial engagement of both otoliths and semicircular canals within the vestibular system (i.e., ‘vestibular actions’), while actions limited to the upper limbs do not require head or trunk movements and thus entail minimal vestibular activity (i.e.; ‘non-vestibular actions’). With *familiarity* we refer to the extent of motor expertise about the action, derived from its frequency in daily execution. To check for the motor familiarity of the action, stimuli were previously validated by administering a Familiarity Questionnaire (see below). A total of four action categories were finally identified: (1) familiar non-vestibular actions, (2) non-familiar non-vestibular actions, (3) familiar vestibular actions and (4) non-familiar vestibular actions. For each category, there were three variations of the same action differing in minor details. A total of 24 videoclips were generated, combining different factors: familiarity (familiar, unfamiliar), vestibularity (vestibular, non-vestibular), gender (male, female), and variations (1, 2 and 3). Finally, an image representing the climax of the action was extracted for each clip (see Supplementary Materials).

### 2.3 Action discrimination task (ADT)

**Fig. 1:**
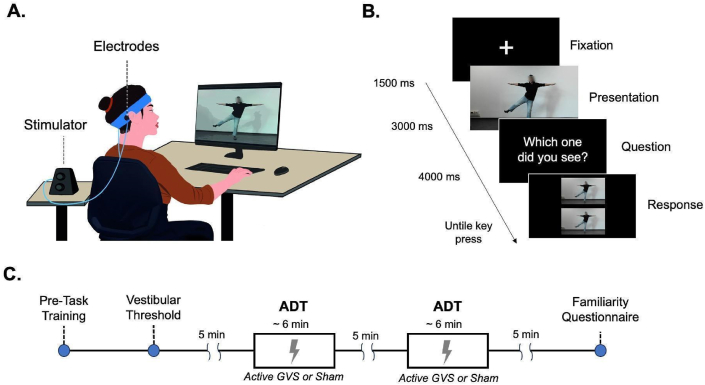
(A). Experimental setting and GVS montage (B) Timeline for each trial of the Action Discrimination Task; (C) Timeline of the experimental session.

In the ADT we used a two-alternative forced choice (2AFC) method. Participants were instructed to fixate on a central cross on the screen for 1500 ms without moving their eyes. A 3-s video depicting a specific action was then shown (*the presentation phase*), followed by a black screen displaying the question “Which one did you see?“. After a delay of 1000 ms, two images were displayed vertically with one corresponding to the climax of the previously viewed video. Participants were required to choose one of the two options by pressing, with their dominant hand, either the up or down arrow keys on the keyboard (*the response phase;* see Fig. 1B). Both the correct and the incorrect choices represented actions performed by actors other than those shown in the target video to mitigate potential confounding factors that could arise from the correspondence between the actor's physical attributes and kinematics. The two images remained on the screen until participants pressed the button and then a new trial started. In each condition, the 24 action videos were presented in a randomized and equally repeated manner to form a battery of 72 trials (i.e., three times for video), lasting about 6 min. The task was created with MonkeyLogic [31], a MATLAB app (MathWork, MA, USA). Response times (i.e., the time interval between the stimulus presentation and the participant's response) and accuracy (i.e., percentage of correct answers) were collected for each trial.

### 2.4 Familiarity questionnaire

The questionnaire was inspired by the one utilized in a previous study testing the motor and visual familiarity with the observed actions [11]. Here, three main factors were evaluated: (1) the motor familiarity (question: ‘How often do you perform this movement?‘), (2) the movement complexity (question: ‘Is it difficult to perform this movement in general?‘) (3) the subjective reproducibility (question: ‘Are you able to replicate this movement?‘). For each stimulus, participants were asked to give a rating from 1 (little) to 5 (very much) to all the three questions.

### 2.5 Galvanic Vestibular stimulation

Supra-threshold GVS was delivered using a stimulator (Soterix Medical, Inc., New Jersey, USA) that provided direct current (DC) with low-frequency sinusoidal waveforms (1 hz) to carbon rubber electrodes placed over the mastoid processes (Fig. 1A). The vestibular threshold was determined for each participant as the minimum stimulation level that evoked a clear and consistent sensation of movement [32,33]. Electrodes were coated with conductive gel to ensure skin comfort and electrical continuity and were secured using a holder and elastic strap for consistent contact with the mastoid. Participants were instructed to stand upright with arms extended alongside their body and eyes closed. Then, starting with a low-intensity DC stimulation (.1 mA), the experimenter gradually raised the intensity using the sliding bar of the stimulator until the participant subjectively reported a swaying sensation and the swaying was objectively visible to the experimenter. Afterward, the intensity of the stimulator was gradually decreased to zero. Once the stimulation was over, participants were asked to reopen their eyes and move their limbs to regain confidence in their body position and movements. This procedure was repeated 3 times, and the average of the three trials was taken as the vestibular threshold. The stimulation protocol was bipolar and binaural, with left-anodal and right-cathodal configuration named ‘L-GVS’ or left-cathodal and right-anodal configuration named ‘R-GVS’. Participants were randomly assigned to one of two groups. Each group, with the assigned montage, also underwent a Sham-GVS. The sham condition was designed to mimic the initial skin sensation of active-GVS, and consisted of the same electrode montage, but with a current that ramped up for 10 s, and then ramped down for 10 s, without delivering any real stimulation.

### 2.6 Experimental procedure

Participants received pre-task training to familiarize themselves with the ADT and with GVS sensations. Once the individual threshold was established while standing, participants were instructed to sit comfortably in front of the laptop with their eyes open to initiate the first assigned condition (i.e., active-GVS or Sham-GVS). The task started once the stimulation amplitude had reached the individual threshold of the participant (in active-GVS) or after the ramp was over (in Sham-GVS) and lasted until participants completed the task (around 6 min). During the experiment, any visible movement or swaying of the participants' heads and bodies was monitored by the experimenter, who had to note the direction and time of the movement or swaying. The order of active GVS and Sham conditions was balanced within each group (R-GVS and L-GVS) in a single-blind fashion. At the end of the first condition and after a 5-min break, another block was repeated under the opposite condition. Following the end of the task and a subsequent break, participants completed the Familiarity Questionnaire. Overall, the whole experimental procedure lasted about 40 min (Fig. 1C).

### 2.7 Statistical analysis

Participants with an ADT accuracy lower than 50 % in sham condition were excluded from the analysis. Differences between the two groups were analyzed using Kruskal-Wallis test for continuous variables (i.e., age, sport frequency, threshold).

*Response Times.* To obtain comparable measures among the participants, ADT response times (RTs) in ADT were first intra-subject normalized using z-score transformations The Shapiro-Wilk test, performed on the z-transformed values, indicated that all variables were normally distributed*.* A repeated-measures ANOVA was performed using Group (*L-GVS, R-GVS*) and Sex (*Male, Female*) as between-subjects’ factors and Stimulation (*Sham, GVS*), Familiarity (*Familiar, Non-Familiar*) and Vestibularity (*Vestibular, Non-Vestibular*) as within-subjects factor. Post-hoc comparisons were then performed using a series of paired t-tests with Bonferroni correction.

*Signal detection analysis.* To further analyze perceptual sensitivity and response biases in the ADT, we employed the Signal Detection Theory [34], a widely-used methodology that allows to disentangle an observer's ability to discriminate between signal and noise (sensitivity, quantified as *d'*) from their criterion tendency to favor one response over the other (response bias, quantified as *c*) for our 2AFC Match-to-Sample Task. For each action, we quantified the Hit Rate (HR), defined as the proportion of correct responses for an action during the response phase, when the action was presented in the presentation phase. We also assessed False Alarms (FA), representing the proportion of responses for the same action in the response phase, when it was not presented during the presentation phase. Extreme proportions of HR and FA (i.e., 0 % and 100 %) were corrected using a log-linear approach [35]. Participants' d’ values were computed by subtracting z-scores of FA from z-scores of HR. On the other hand, c values were computed as the negative average of the z-transformed HR and FA. Positive c values indicate a bias towards a more conservative criterion, whereas negative values indicate a more liberal criterion in the action response. Such parameters were computed separately for the action category (Familiar/Unfamiliar) and the stimulation condition (Sham/GVS) with the Matlab Palamedes Toolbox [36]. The Shapiro-Wilk test indicated that all variables were not normally distributed. The nonparametric Wilcoxon signed-rank test was then performed for each action category to compare *d'* and *c* values between GVS and Sham conditions. Correlations between *d', c*, and RTs for each action category during both active-GVS and sham-GVS were also calculated using Spearman's correlation. Statistical significance of *p* < .05 was assumed. All the analyses were performed using SPSS Statistics software (IBM, version 28.0).

*Familiarity questionnaire.* To control for motor familiarity with action stimuli, the Wilcoxon signed-rank test was performed to compare Familiarity Questionnaire ratings between the four action categories.

## 3 Results

### 3.1 Groups

Seven participants were excluded from the analysis because of the low accuracy score in the task during Sham-GVS (i.e., <25 %). A final sample of 25 participants (11 females and 14 males, mean age 24.8 ± 1.9), 12 in L-GVS and 13 in the R-GVS group, were included in the analysis. Importantly, the two groups (i.e., L-GVS and R-GVS) did not differ for age [z = -.28, *p = .77*] and stimulation threshold [z = −.10, *p = .91;* mean = *L-GVS group:* 1.23 ± 1.5 mA*, R-GVS group:* 1.24 ± .4 mA]. The GVS was well tolerated by all participants and no one showed head or body sways during the experiment. The demographic characteristics and stimulation parameters for each participant are reported in Table 1.

**Table 1:** Demographic data and stimulation parameters.

Subjects	Sex	Age	Sport Frequency	Sport types	Anodal Side	Threshold (mA)	Order
**1**	M	26	2 times/week	Martial arts	Right	1.07	A-S
**2**	M	24	2 times/week	Running	Right	.49	S-A
**3**	F	22	Rarely	Dance	Left	.45	A-S
**4**	M	25	2 times/week	Swimming	Left	.65	S-A
**5**	F	25	2 times/week	Cycling	Right	1.17	S-A
**6**	M	27	1 times/week	Swimming	Right	1.34	A-S
**7**	M	27	Never	/	Right	1.21	A-S
**8**	M	24	Never	/	Right	1.3	A-S
**9**	F	24	2 times/week	Aerial silk	Left	.66	S-A
**10**	F	27	Rarely	Gym	Left	.6	A-S
**11**	F	23	3 times/week	Crossfit	Right	1.08	S-A
**12**	M	23	1 times/week	Boxe	Left	1.25	A-S
**13**	M	24	3 times/week	Boxe	Left	1.83	A-S
**14**	M	30	3 times/months	Football	Left	1.77	A-S
**15**	F	24	3 times/week	Gym	Left	1.15	A-S
**16**	M	24	2 times/week	Boxe	Left	1.76	A-S
**17**	F	24	2 times/week	Yoga	Right	.55	A-S
**18**	F	24	3 times/week	Cycling	Right	.85	S-A
**19**	M	27	2 times/week	Tennis	Right	1.92	S-A
**20**	F	24	1 times/week	Gym	Left	1.28	S-A
**21**	F	24	3 times/week	Gym	Right	1.62	A-S
**22**	M	24	2 times/week	Volleyball	Right	1.93	A-S
**23**	F	22	3 times/week	Gym	Right	1.59	S-A
**24**	M	27	3 times/week	Triathlon	Left	1.97	S-A
**25**	M	27	3 times/week	Gym	Left	1.47	S-A

A: Active GVS; S: Sham.

### 3.2 Action discrimination task (ADT)

*Response times.* A repeated-measures ANOVA on RTs showed a main effect of *Familiarity* [F_(1,21)_ = 86.37, *p* < *.001;* η_p_^2^ = .80] and *Vestibularity* [F_(1,21)_ = 4.56, *p = .045;* η_p_^2^ = .17]. Participants were faster in discriminating familiar actions than unfamiliar actions independently of vestibularity. Participants were also faster in responding to actions without vestibular components than actions involving the vestibular system independently of their familiarity. The interactions *Vestibularity x Familiarity* [F_(1,21)_ = 133.01, *p* < *.001;* η_p_^2^ = .864], *Vestibularity x Familiarity x Group* [F_(1,21)_ = 5.880, *p = .024;* η_p_^2^ = .219] and *Stimulation x Vestibularity x Familiarity* [F_(1,21)_ = 7.209, *p = .014;* η_p_^2^ = .256] were also significant. A post-hoc paired *t*-test on the *Vestibularity × Familiarity* interaction showed that the familiarity effect was significantly smaller for vestibular actions than for non-vestibular actions [*Vestibular*: .12 ± .37, *NonVestibular*: 1.3 ± .55; t_(24)_ = -11.09; *p* < *.001;* d = 2.21], suggesting a minor familiarity advantage on action identification for vestibular actions. An independent *t*-test on the *Vestibularity x Familiarity × Group* interaction showed faster RTs for vestibular unfamiliar actions in the R-GVS group compared to the L-GVS group [t_(23)_ = 2.800; *p = .010;* d = 1.12]. This finding indicates a difference between the two groups in the recognition time of vestibular unfamiliar actions, irrespective of vestibular stimulation. No other comparisons between groups were statistically significant.

**Fig. 2:**
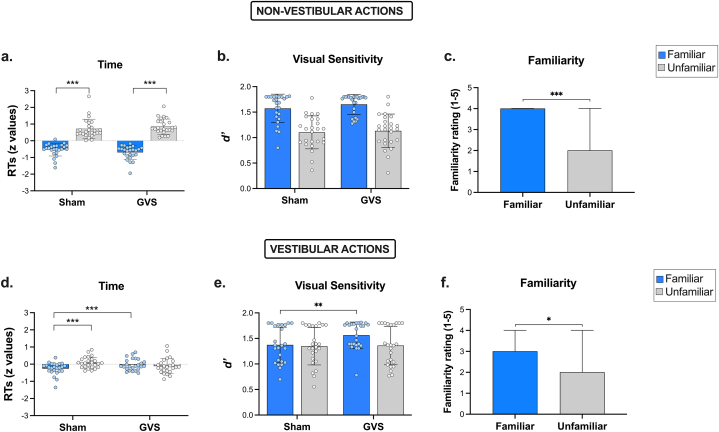
Performance in the Action Discrimination Task for the four action categories. Response times (RT); visual sensitivity (*d’*); subjective motor familiarity for non-vestibular (a, b, c) and vestibular (d, e, f) actions. Data are presented as mean and standard deviation (SD), ∗p < .05, ∗∗p < .01, ∗∗∗p ≤ .001.

Furthermore, post-hoc comparisons with Bonferroni adjustment on the three-way interaction *Vestibularity x Familiarity x Stimulation*, revealed that active-GVS decreased the familiarity effect for vestibular actions compared to Sham-GVS [t_(24)_ = -2.68, *p = .013;* d = .52], but not for non-vestibular action. In particular, participants showed faster RTs for familiar actions compared to non-familiar actions during Sham-GVS, both for non-vestibular [t_(24)_ = -7.06, *p* < *.001,* d = 1.41; Fig. 2a] and vestibular actions [t_(24)_ = -3.07, *p = .005,* d = .61*;* Fig. 2d]. However, during active-GVS, familiar actions were discriminated significantly faster than those unfamiliar only when actions were not related to the vestibular system [t_(24)_ = -11.92, *p* < *.001,* d = 2.38]. Indeed, active-GVS selectively increased RTs in discriminating vestibular familiar actions compared to Sham-GVS [t_(24)_ = 2.93, *p = .004,* d = .59], leading to the disappearance of the familiarity effect for actions with vestibular component (Fig. 2d). Following exclusion criteria, we excluded ten participants from the analysis. Thus, we decided to run an effect-size sensitivity analysis conducted with G∗Power [29], to test if our final sample size is adequate to detect the effect-size of our critical comparison (i.e., the effect of GVS on familiar action discrimination) [37]. The results revealed that, considering our final sample size (n = 25), alpha (.05), and 80 % of power, we have the sensitivity to detect an effect size dz = .58 in a two-tail paired *t*-test. Such effect is comparable with the one we found in our critical comparison (dz = .59), indicating that our sample size is appropriate for detecting the observed effect size with adequate power and supporting the reliability of the findings.

*Signal detection analysis.* The Wilcoxon signed-rank test revealed statistically significant differences between *d'* values of familiar vestibular actions when comparing Sham-GVS and active-GVS [z = −2.128; *p = .03,* r = .21]. In particular, *d'* mean values were higher in active-GVS than in Sham-GVS (Fig. 2e), indicating that stimulation selectively increased the discriminability of familiar vestibular actions. No other comparisons between conditions were statistically significant. The Wilcoxon signed-rank test revealed also no statistically significant differences between *c* values’ conditions (p > .05).

**Fig. 3:**
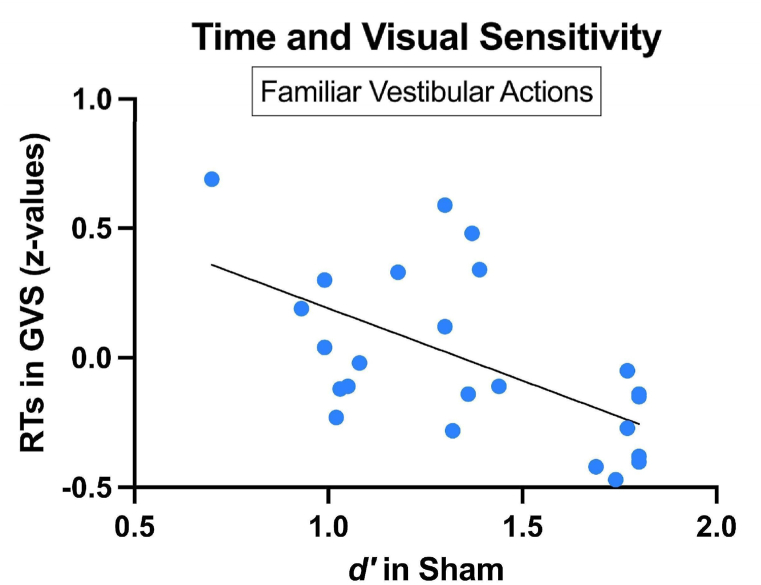
Spearman's correlation between individual *d'* during Sham-GVS and response times during active-GVS for familiar vestibular actions.

*Correlation analysis*. A set of Spearman correlations was performed to analyze the relationships between *d'*, *c*, and RTs for each action category during both sham-GVS and active-GVS. A significant negative correlation was found between *d'* in sham-GVS and RTs during active-GVS, uniquely for vestibular familiar actions [r = −.059; *p = .002*]. More specifically, higher *d'* values in the sham-GVS were associated with faster RTs during active GVS (Fig. 3), indicating that participants showing higher discriminability during the sham condition also showed faster RTs under the influence of GVS.

### 3.3 Familiarity questionnaire

The Friedman test revealed a statistically significant difference on motor familiarity ratings depending on action category [χ^2^ (3) = 22.339; *p* < *.001*; w = .298]. The post-hoc Wilcoxon signed-rank test showed that ratings for familiar stimuli were significantly higher than those unfamiliar, both for non-vestibular [z = −4.327, *p* < *.001*; r = .86; Fig. 2c] and vestibular actions [z = −2.309; *p = .021*; r = .46; Fig. 2f]. Furthermore, familiar non-vestibular actions were rated as more familiar than familiar vestibular actions [z = −3.470; *p* < *.001;* r = .69].

## 4 Discussion

Previous studies have shown that the identification of others’ actions is mediated by the activity of mirror mechanisms, which strongly involve the motor system [5]. Despite the well-known role of the vestibular system in the execution and perception of complex self-movements, its possible contribution to the construction of sensorimotor representations underlying the identification of others' actions is still unknown. To fill this gap, we investigated the impact of supra-threshold GVS on the discrimination of actions that may or may not require vestibular activity to be performed. Since motor familiarity can play a crucial role in action identification, we examined its impact on vestibular and non-vestibular actions (i.e., familiar vs unfamiliar). The results show that the modulation of the vestibular signal induced by GVS selectively interferes with the identification of vestibular actions that were also familiar. In other words, the familiarity advantage observed in Sham-GVS disappeared for vestibular actions during active-GVS. Interestingly, active-GVS not only increased response times for the identification of vestibular familiar actions but also improved the visual sensitivity for these specific actions. These seemingly counterintuitive results reveal the complex interaction between the vestibular system and visually mediated action perception and show the contribution of the vestibular system in mirror processing during action identification, as discussed in the following paragraphs.

### 4.1 Effect of familiarity on action recognition response times

As expected, results showed faster response times in recognizing familiar actions as compared to unfamiliar ones. This advantage was observed regardless of whether the action was vestibular or not. Differences in response times between familiar and unfamiliar actions are likely to reflect the advantage given by motor familiarity, which would accelerate the action identification process through the activation of more accurate sensorimotor representation. The observation of an action activates internal models that are typically used for the execution of the same action, thus enabling the simulation of motor commands and the prediction of their outcomes [38]. The more familiar the observer is with the execution of this action, the more accurate is the sensorimotor representation generated by its internal model, resulting in less prediction error and faster recognition of the action. Consistently, in our study non-vestibular actions were rated as more familiar and recognized more quickly than vestibular ones. While previous studies reported the motor familiarity advantage during action identification on neural activity [39], visual sensitivity [11] and action prediction [14], to the best of our knowledge, only a few studies extended this effect to response times [15,40]. These results confirm that response times reflect the degree of motor familiarity with the observed actions and support the existing literature that emphasizes improvement in action recognition due to motor expertise.

### 4.2 Differential impact of GVS on response times and visual sensitivity

Consistent with our hypothesis, active-GVS selectively interfered with the recognition of actions with strong vestibular components. This interference was observed for familiar but not for unfamiliar vestibular actions, in line with the evidence that the observation of familiar actions elicits more accurate sensorimotor representations than those unfamiliar [32] and thus they might be more sensitive to detect GVS interference on mirror-like processing.

Surprisingly, GVS, on the one hand, selectively slowed down response times in the recognition of vestibular familiar actions and, on the other hand, increased participants' visual discriminability (or sensitivity, as measured by *d'*) for the same actions. Although counterintuitive, these results align with theoretical frameworks of predictive coding of action identification and suggest a direct contribution of the vestibular system in mirror processing [41,42]. When observing a familiar action, the brain uses internal models based on past experiences to simulate the corresponding motor command and generate predictions about future trajectories and outcomes of the observed action. Slower response times when discriminating vestibular familiar actions during active-GVS indicate that disruption of vestibular nerve firing interfered with the simulation of the sensorimotor representation of actions requiring vestibular input. On the other hand, the increase in visual sensitivity suggests that, when the sensorimotor representation of observed actions is impaired, individuals rely more on visual features of body parts [43]. Notably, the two available action choices presented during ‘the response phase’ were different only for subtle variations in limb orientation. Greater visual sensitivity indicates a greater ability to visually discriminate these details, revealing a greater reliance on the processing of body parts during action identification. This modality of stimuli processing is compatible with the observed increase in response times.

Over the last decades, two pathways have been proposed to explain two components of action identification: the meticulous analysis of local parts of the body and the holistic processing of global movements [[43], [44], [45]]. The first network, the *ventral pathway*, involves the extrastriate-body area (EBA) that, within the visual domain, dissects individual body elements and focuses on specific details such as limb positions and orientations. A second network, the *dorsal pathway*, includes frontoparietal regions such as the ventral premotor cortex (vPMc), IPL and STS, crucially involved in sensorimotor and mirror processing. This network would encode global sensorimotor representations enabling us to rapidly discern postures and movements in their entirety. Interestingly, empirical evidence has demonstrated that vestibular stimulation activates areas within the *dorsal pathway*, such as the IPL and STS [23,46]. It can therefore be speculated that the vestibular stimulation administered in this study selectively disrupted the activity of the *dorsal pathway*, which is specialized for *sensorimotor representation*, and that this disruption increased the weight of the *ventral pathway*, which is mainly responsible for *visual representation*. This speculation could offer a possible explanation for the observed decrease in response times and increase in visual sensitivity for vestibular familiar actions induced by GVS. Consistently, the negative correlation observed between visual sensitivity shown during sham-GVS and response times during active-GVS aligns with this interpretation. Participants with a stronger reliance on visual representations without stimulation exhibited faster response times during vestibular stimulation, indicating a minor impact of GVS in individuals relying more heavily on local visual features for action identification. These findings are in line with existing evidence that when vestibular signals are impaired or diminished, the weight of the visual information is increased and the brain mainly relies on visual information for spatial processing [[47], [48], [49], [50], [51]].

These results reveal differential effects of GVS on action identification, depending on the extent to which visual or sensorimotor representations are engaged in the identification process.

### 4.3 General discussion

The critical involvement of vestibular signals in action execution and self-motor perception has been extensively documented [18]. We investigated, for the first time, the causal role of vestibular function in recognition of others' action, by applying disruptive GVS [52], as is done with TMS using inhibitory or interference protocols [[53], [54], [55]]. Interestingly, our findings show that the vestibular system is also causally implicated in the perception of actions performed by others. This result is consistent with a recent proposal about the existence of a *vestibular mirror system* [56]. Lopez and colleagues [56] suggested that the vestibular perception is modulated by agent-specific mirror mechanisms, as observing someone else in motion influences self-motion perception. However, despite yielding compelling results, our interpretations should be taken with caution due to the lack of neuroimaging data. Indeed, while previous neuroimaging research has delineated the involvement of the ventral pathway in recognizing specific features of actions and the dorsal pathway in a more global sensorimotor representation [36], our investigation primarily focused on the brain interference obtained with peripheral vestibular stimulation and relative behavioral consequences. Future neuroimaging studies are needed to support the hypothesis that modulation of brain activity induced by GVS distinctly targets regions within the ‘dorsal pathway’ and whether these modulations correlate with behavioral effects similar to those unveiled by the present study. It would also be relevant to evaluate whether altered action identification such as that induced by transient GVS interference can be observed in patients with acute vestibular disorders and/or patients with chronic vestibular disorders after experiencing prolonged difficulties in performing vestibular actions. Our study employed a single-blind research design, where participants were unaware of the stimulation condition they were undergoing (active L-anodal, active R-anodal, or non-active sham). Although this design adequately controls for potential placebo effects, future GVS studies must employ double- or triple-blind designs, to further minimize biases related to the experimenters' expectations and enhance the reliability of the findings. These designs ensure that besides the participants both the experimenters administering the study and the ones carrying out the statistical analyses are unaware of the conditions they are administering or analyzing, thereby significantly improving the reliability of the findings. Finally, although our analysis showed adequate statistical power, the groups size were comparatively small and future studies with larger sample size are necessary to replicate and validate these new findings.

Beyond theoretical interpretations and methodological limitations, the observed modulation induced by GVS in recognizing action reliant on vestibular activity may have important implications, particularly for individuals with vestibular or sensorimotor disorders. For example, employing noisy stimulation protocols may harness the potential of GVS and offer new avenues for enhancing action perception and motor function. This technique administers low levels of stochastic electrical stimulation to the afferent vestibular nerve, increasing the detectability of subthreshold signals and thus the sensitivity to detect motion and orientation changes [57]. Prior evidence suggested that noisy GVS may improve dynamic locomotion in healthy individuals [[58], [59], [60]] and in patients suffering from vestibular [[61], [62], [63], [64]] or neurodegenerative disorders [[65], [66], [67], [68], [69], [70], [71], [72]]. Studies in the field of space cognitive neuroscience have used GVS to develop countermeasures for adaptation to zero gravity [73,74] as the unloading of the vestibular organs in microgravity or ground-based analogs significantly affect sensorimotor function [74], somatosensory perception [[75], [76], [77]], and spatial attention [51]. However, future studies using randomized controlled trials and noisy GVS protocols are needed to investigate the effect of different stimulation parameters, including intensity, duration, and frequency, to optimize its application for therapeutic purposes.

## 5 Conclusions

In conclusion, the results of this study show that the vestibular system, which is known to be involved in action execution, also contributes to the perception of the actions of others. The vestibular stimulation may selectively disrupt the sensorimotor processing carried out by the dorsal pathway implicated in the observation of actions, resulting in the alteration of the mirror process. Consequently, this modulation may prompt individuals to increasingly rely on a second processing system based on the perceptual analysis of limb positions. This specific dissociation may offer new valuable insights for future investigations about the intricate relationship between sensory inputs and cognitive processes involved in action identification. Future studies are needed to understand the role played by vestibular information in action representation, in order to develop innovative and personalized approaches to substantiate the effectiveness of GVS-based interventions in ameliorating sensorimotor functioning and enhancing the quality of life in individuals dealing with sensorimotor or vestibular disorders.

## Data availability statement

All data are available at the Mendeley Data Repository and can be accessed at

https://data.mendeley.com/preview/zdrgj8w9jm?a=65048911-7a00-45ab-9548-d0bc77002df1.

## CRediT authorship contribution statement

**Roberto Gammeri:** Writing – review & editing, Writing – original draft, Supervision, Methodology, Formal analysis, Data curation, Conceptualization. **Maria-Chiara Villa:** Writing – review & editing, Writing – original draft, Supervision, Methodology, Formal analysis, Data curation, Conceptualization. **Tommaso Ciorli:** Writing – review & editing, Formal analysis, Data curation. **Anna Berti:** Writing – review & editing, Conceptualization. **Raffaella Ricci:** Writing – review & editing, Supervision, Data curation, Conceptualization.

## Declaration of competing interest

The authors have declared no conflict of interest.
