# Supplementary material for: Beyond balance: The role of the Vestibular system in action recognition
Source: Heliyon. 2024 Sep 18;10(18):e38019. doi: 10.1016/j.heliyon.2024.e38019 (PMC11438003; doi:10.1016/j.heliyon.2024.e38019)

**Supplementary Materials.** Stimuli presented in the ‘Response Phase’ of the Action Discrimination Task. Each image represents the climax of the action presented in the ‘Presentation Phase’, which could be: (1) familiar non-vestibular actions, (2) non-familiar non-vestibular actions, (3) familiar vestibular actions and (4) non-familiar vestibular actions. For each category, there were three variations of the same action, differing in minor details. Furthermore, each action was performed by either a male or a female.

Non-Vestibular

*Familiar*


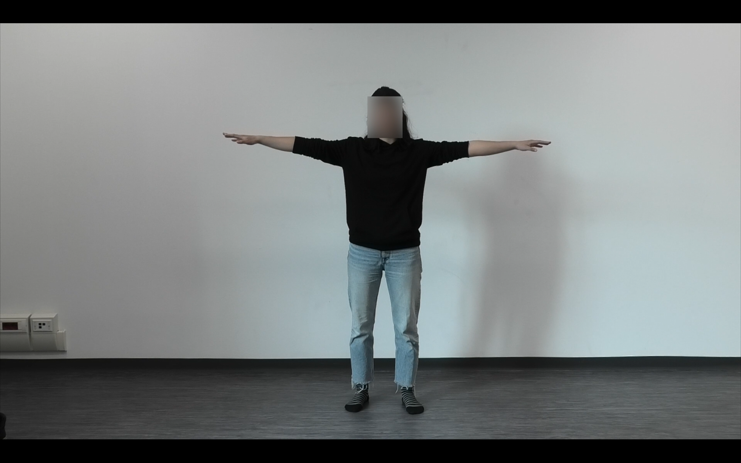

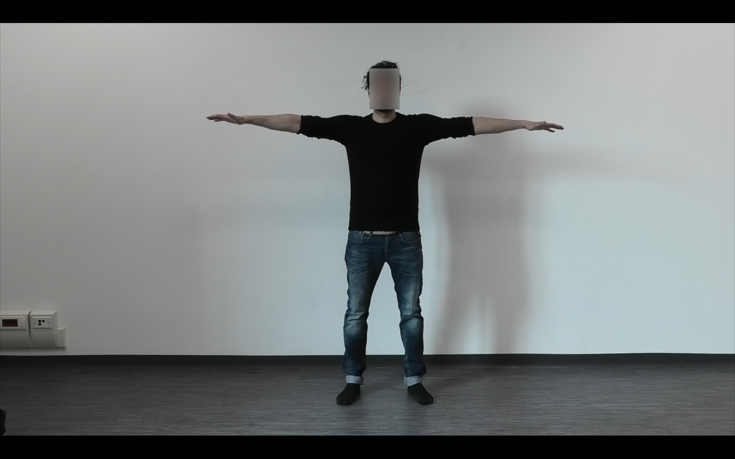


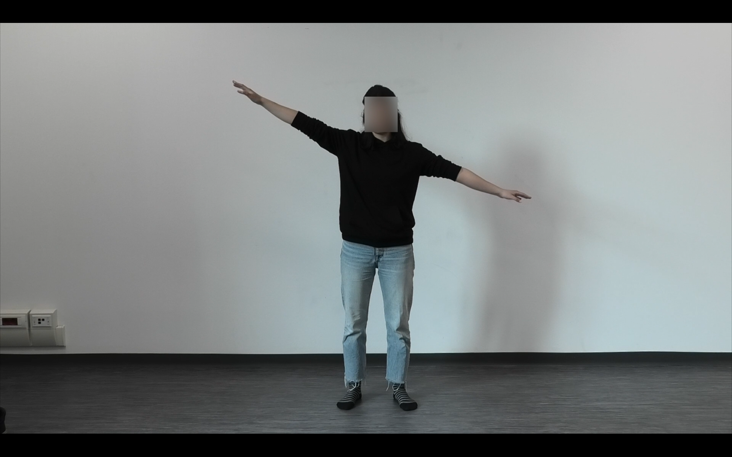

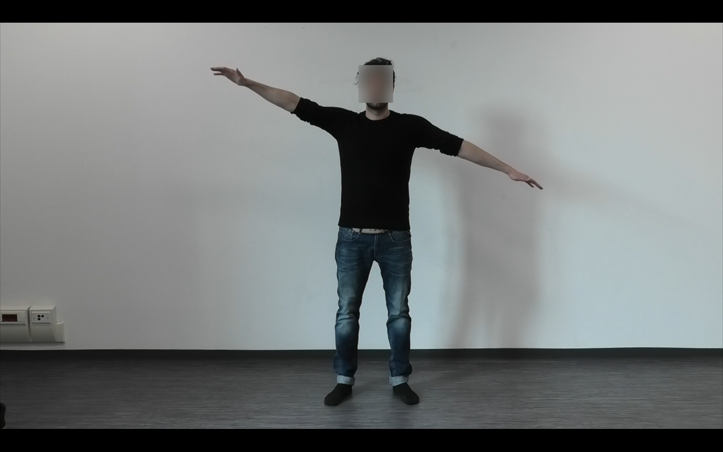

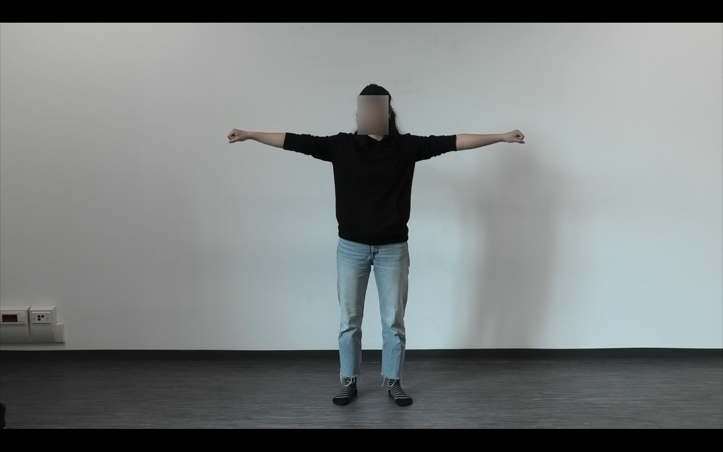

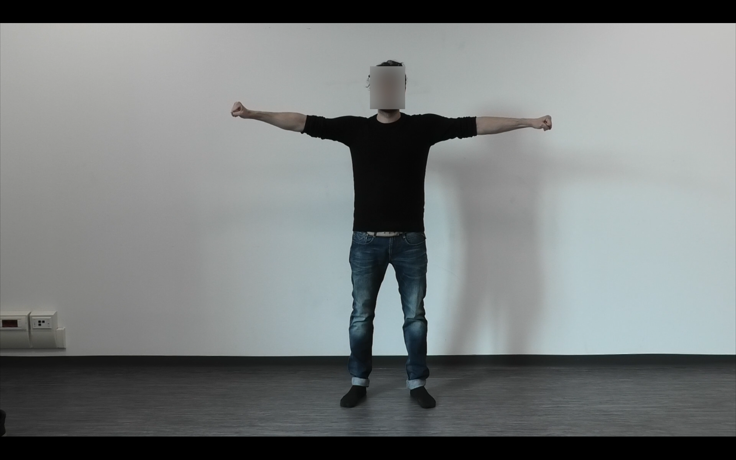


*Unfamiliar*


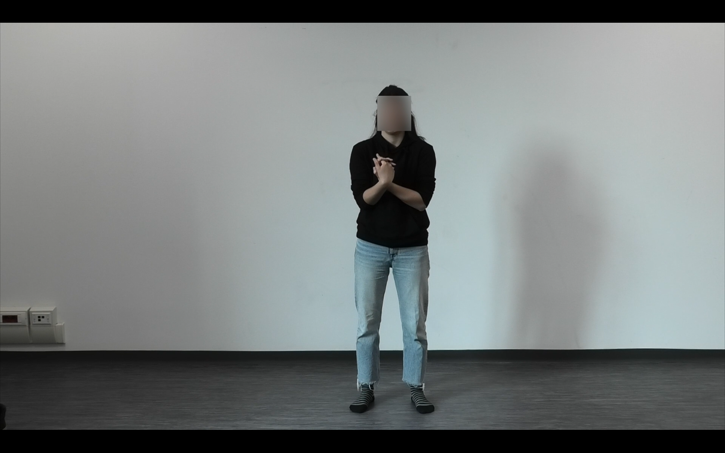

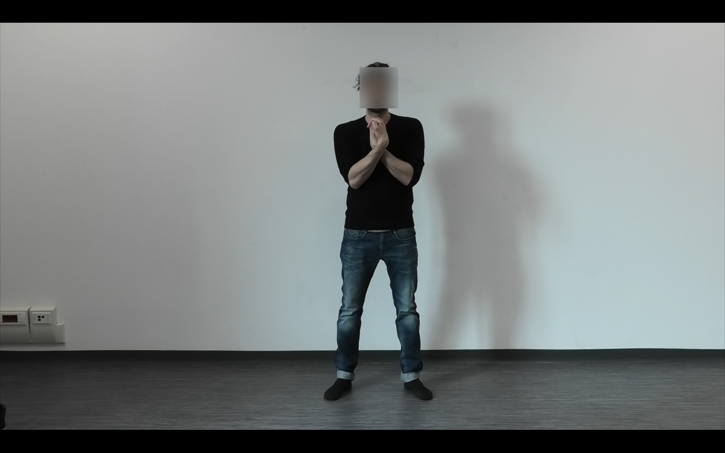

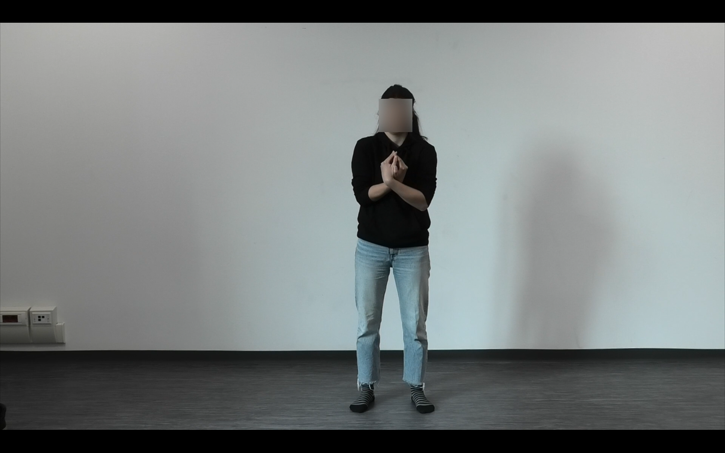

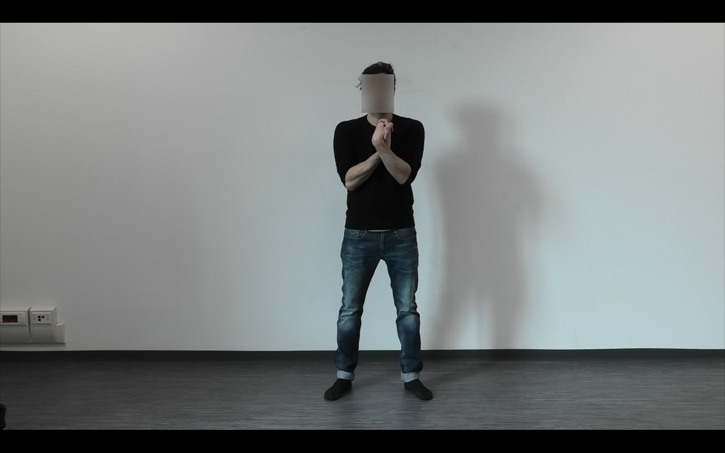


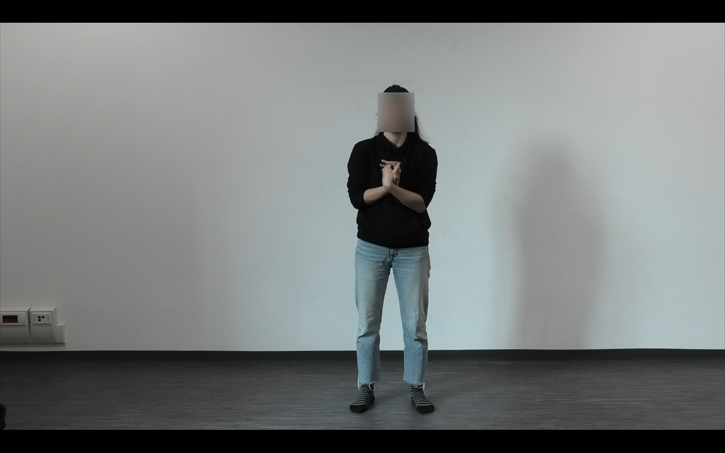

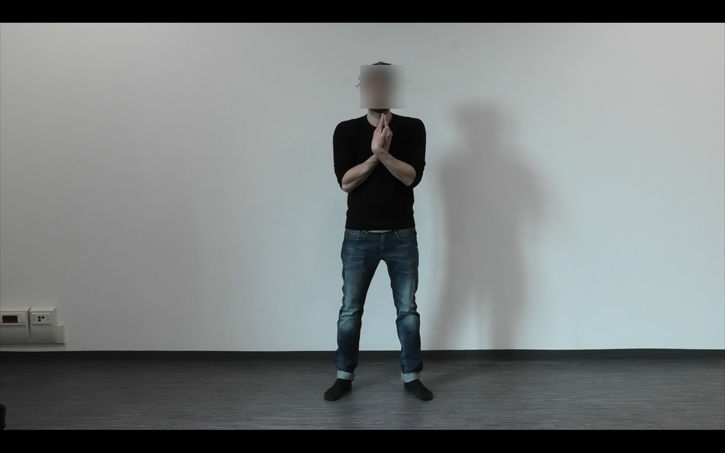


Vestibular

Familiar


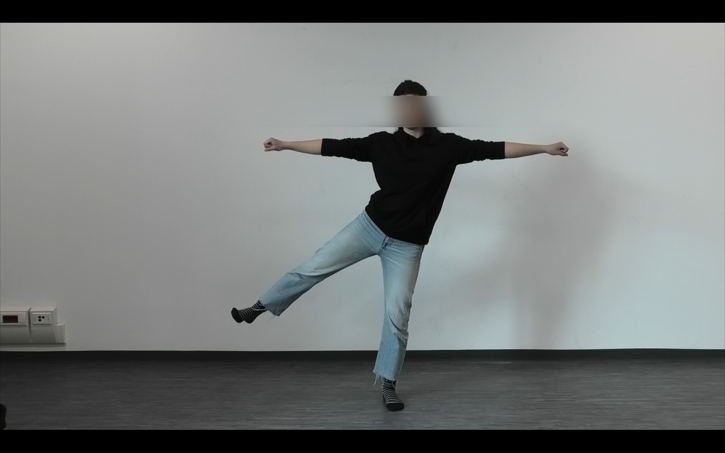

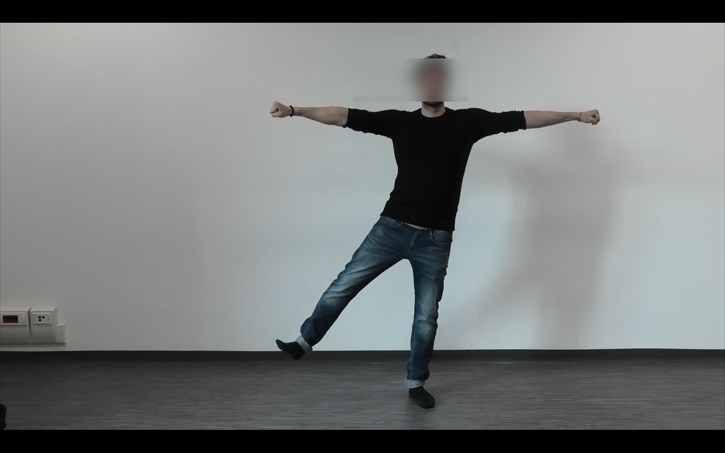


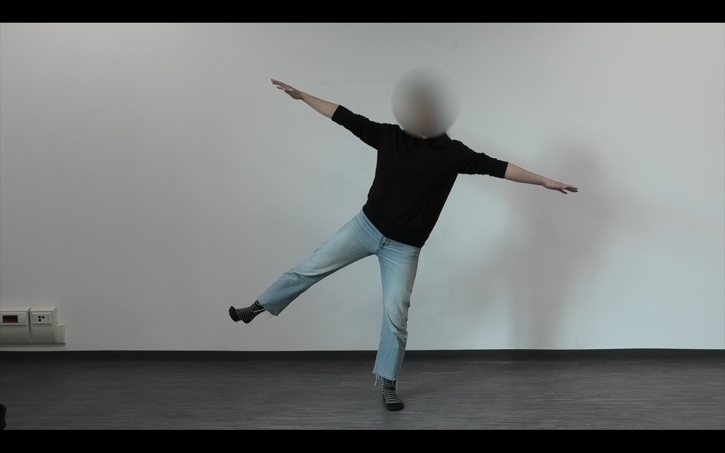

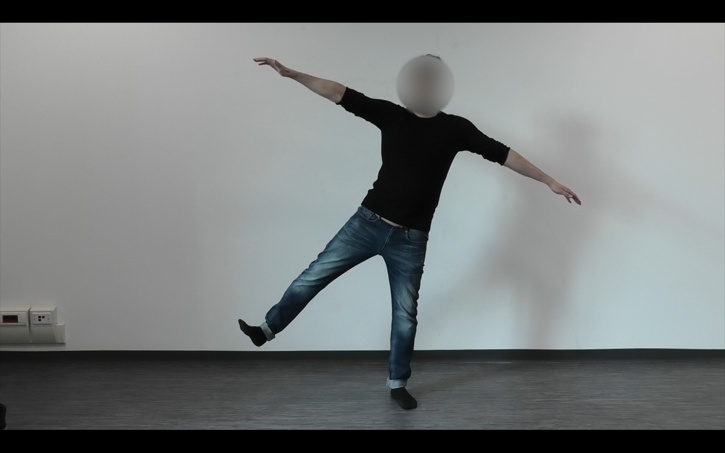


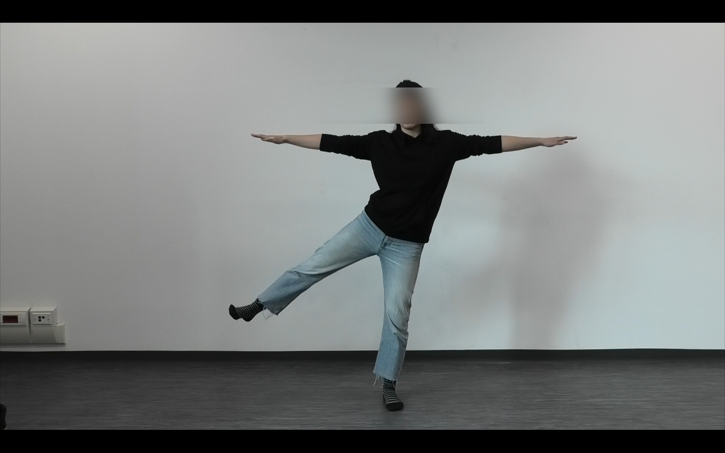

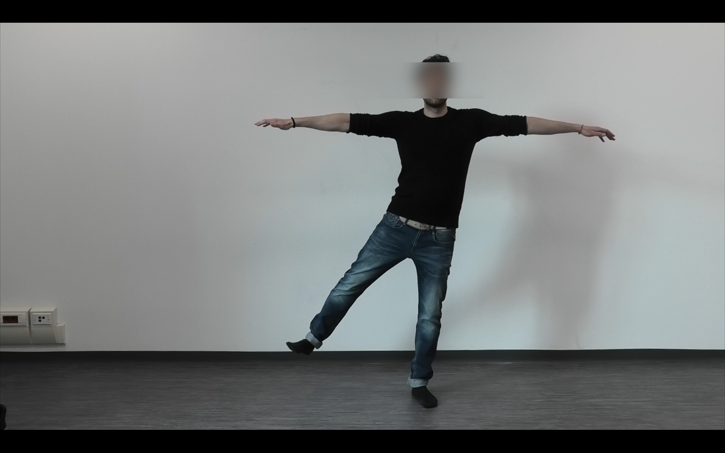


*Unfamiliar*


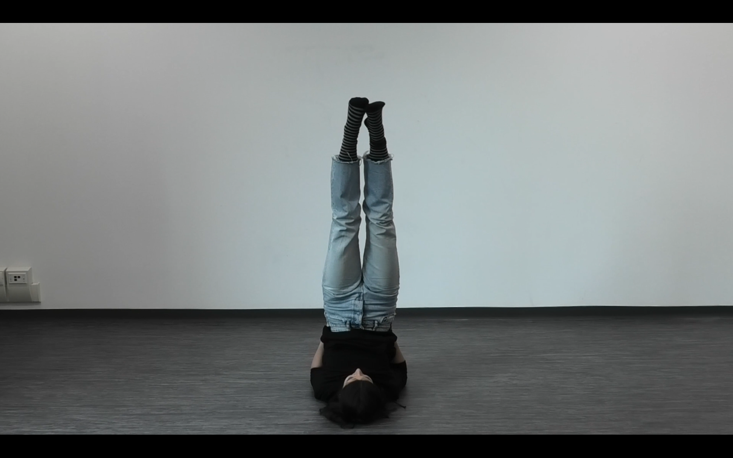

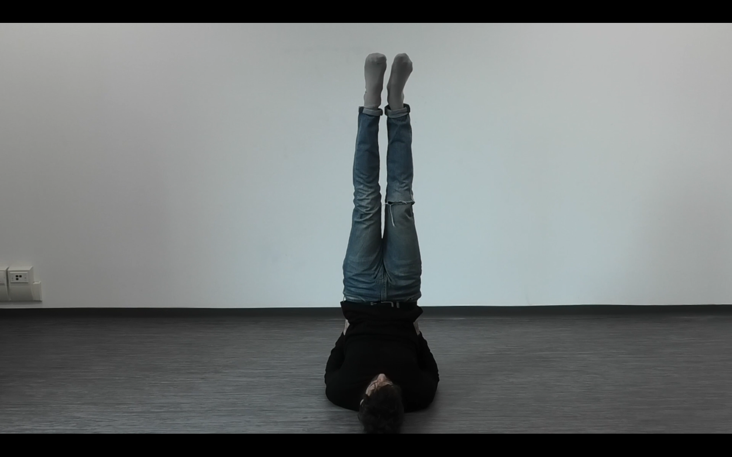

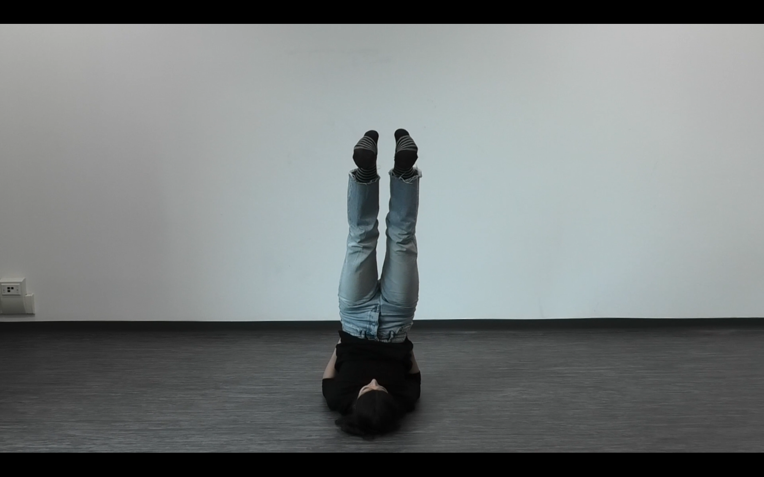

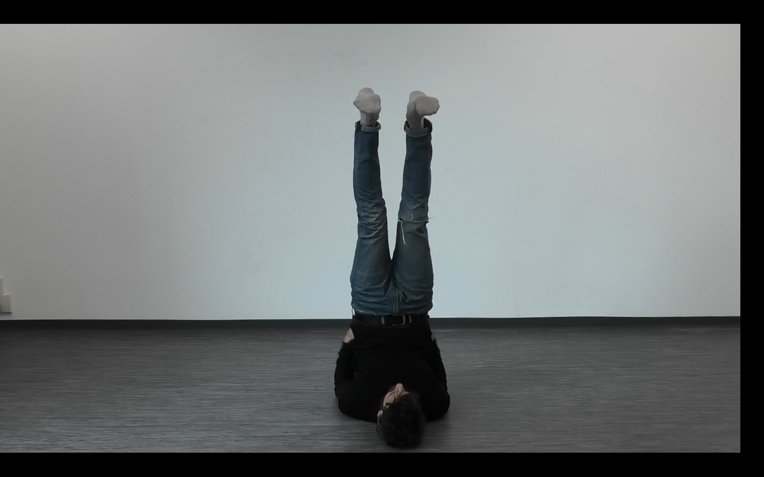

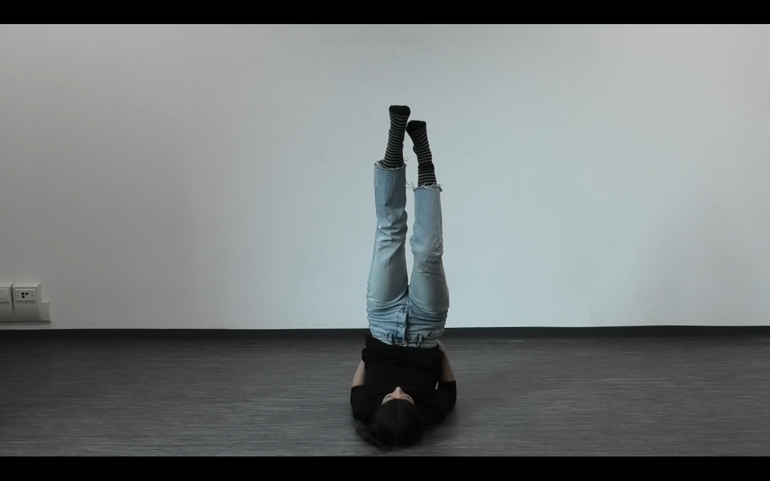

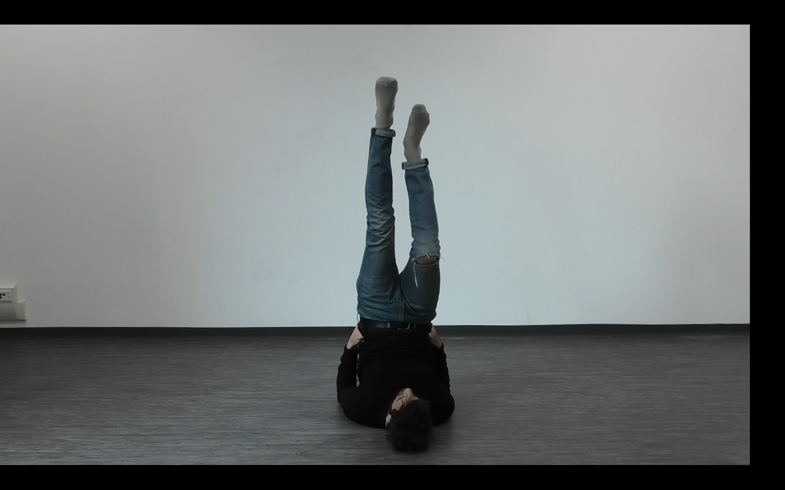

Supplement: Multimedia component 1 [file mmc1.docx]
